# A Role for Yeast/Pseudohyphal Cells of *Candida albicans* in the Correlated Expression of *NLRP3* Inflammasome Inducers in Women With Acute Vulvovaginal Candidiasis

**DOI:** 10.3389/fmicb.2019.02669

**Published:** 2019-11-15

**Authors:** Elena Roselletti, Claudia Monari, Samuele Sabbatini, Stefano Perito, Anna Vecchiarelli, Jack D. Sobel, Antonio Cassone

**Affiliations:** ^1^Medical Microbiology Section, Department of Medicine, University of Perugia, Perugia, Italy; ^2^School of Medicine, Wayne State University, Detroit, MI, United States; ^3^Polo d’Innovazione di Genomica, Genetica e Biologia, University of Siena, Siena, Italy

**Keywords:** *Candida albicans*, acute vulvovaginal candidiasis, vaginal epithelial cells, *SAP2*, *ECE1*, yeast/pseudohyphal cells, *NLRP3* inflammasome

## Abstract

In acute vulvovaginal candidiasis (VVC), the fungus *Candida albicans* activates inflammasome receptors of vaginal epithelial cells through the production of virulence and immuno-inflammatory factors. Here, we show that in VVC patients, genes encoding some of the above factors (*SAP2*, *SAP5*, *SAP6*, *ECE1*, and *HWP1*) are expressed in a correlated fashion. Cytological observations pointed out that pseudohyphal filaments with yeast cells are dominant at the acidic vaginal pH, and this is coupled with co-expression, at roughly similar level, of *SAP2*, a typical yeast and *ECE1*, a typical hyphae-associated genes. In contrast, vigorous hyphal growth dominated at the neutral vaginal pH of mice experimentally infected with *C. albicans* isolates from VVC subjects, and this is coupled with a high ratio of *ECE1* to *SAP2* expression. We suggest that the pseudohyphal rather than true hyphal cells of *C. albicans* play a critical role in VVC, possibly through the activity of multiple inflammasome inducers.

## Introduction

Vulvovaginal candidiasis (VVC) is an infection affecting millions of women worldwide, with 4–8% suffering from disease recurrences (RVVC) that substantially reduce their quality of life (Sobel, 1992, 2016; Denning et al., 2018). Pharmacological control of VVC recurrences, though possible with maintenance antifungal therapy, remains problematic and does not eradicate risk of future infection (Sobel, 1992, 2016). Importantly, VVC and RVVC frequently occur in women without any recognizable immunological deficit or predisposing morbidity. For these reasons, VVC has long attracted attention in an attempt to unravel pathogenesis and immune control of the disease, including vaccination (Cassone, 2015; Edwards et al., 2018).

Clinical research and experimental models in rodents have provided some insight into fungal colonization and vaginal inflammation caused by *Candida albicans* (Fidel, 2002; Fidel et al., 2004; Cassone et al., 2016). In particular, recent investigations have suggested that the hallmark of VVC pathology caused by *C. albicans*, in both rodents and humans, could be the induction by fungal components of an intracellular damage-responsive molecular complex called *NLRP3* inflammasome of vaginal epithelial cells (VEC) (Bruno et al., 2015; Pericolini et al., 2015; Roselletti et al., 2017). *NLRP3* activation triggers a cascade of caspase-1 activation, pro-inflammatory cytokine production, and infiltration of neutrophils that are, however, incapable of eradicating infection and actually contribute to pathology (Gabrielli et al., 2016; Yano et al., 2018).

Past data in human VVC (De Bernardis et al., 1990) and more recently in mouse models (Pietrella et al., 2013; Pericolini et al., 2015; Richardson et al., 2018b; Rogiers et al., 2019) point to members of the secretory aspartic proteinase family (Sap) and candidalysin, a recently discovered, *ECE1*-derived and hyphae-associated, fungal toxin (Moyes et al., 2016), as those most studied factors capable of causing pathogenic *NLRP3* inflammasome activation. Nonetheless, their role in VVC remains undefined.

In a recent paper, we showed that *NLRP3* and caspase-1 transcription in VEC of VVC patients is associated with marked upregulation of expression of some genes coding for both yeast (*SAP2*) and hypha- (*SAP5*, *SAP6*, *HWP1*, and *ECE1*) associated factors, suggesting a relationship to the vaginal inflammatory response (Roselletti et al., 2017). However, the number of VVC subjects examined was limited and thereby unsuitable to establish unbiased, genuine statistical correlations. By using an expanded VVC population, we report here on positive correlations in the expression of the above virulence and immuno-inflammatory factors. Comparative observations in mice experimentally infected with VVC isolates of *C. albicans* also suggest for a dominant role of pseudohyphal, rather than true hyphal cells in VVC.

## Materials and Methods

### Subjects

All patients enrolled in this study attended the microbiological diagnostic service of the University Hospital Santa Maria della Misericordia, Perugia (Italy) over the period from February 2016 to September 2016. Full clinical parameters of the women enrolled in the study were described previously (Roselletti et al., 2017). This paper includes the data from 20 patients additional to those previously considered (Roselletti et al., 2017). Briefly, the present group consisted of 40 non-pregnant, non-diabetic women, aged 19–53 years. Each woman was positive for *C. albicans* isolation from a vaginal swab taken from the posterior fornix and presented at least two of the following acute VVC signs and symptoms: vaginal discharge, itching, burning, and dyspareunia. The clinician responsible for VVC diagnosis measured the vaginal pH (in the range 4.5–5.0, as expected in VVC subjects) and excluded other causes of vaginitis or vaginosis by microscopic and clinical criteria. None of the recruited women reported a previous diagnosis of recurrent vulvovaginal candidiasis (RVVC). All women signed an informed consent in accordance with the Declaration of Helsinki. Local Ethical Committee CEAS (Comitato Etico delle Aziende Sanitarie, Umbria, Italy) approval was received for the whole study (VAG1 n. 2652/15). All methods were performed in accordance with the relevant guidelines and regulations.

### Sample Collection From Vulvovaginal Candidiasis Patients

Full details of sample collection procedures were described previously (Roselletti et al., 2017, 2019). Briefly, a vaginal swab was taken from each participant, soaked in 1 ml of saline and plated on CHROMagar™ *Candida* (VWR International P.B.I., Milan, Italy), then incubated at 37°C for 48 h to evaluate the vaginal colonization by *C. albicans*. The fungus was identified by routine methods and confirmed by MALDI-TOF test (Biomérieux S.A., France). Hundred microliter of each sample were also stained with Haemacolor and examined by light microscopy (Olympus) for the presence of neutrophils and *C. albicans* morphology. Subsequently, the vaginal fluid was centrifuged at 3,000 rpm for 10 min, and then cellular fraction was used for gene expression analysis (Roselletti et al., 2017).

### Quantitative Analysis of *SAP2*, *SAP5*, *SAP6*, *ECE1*, *HWP1*, *KEX2*, *NLRP3*, and *CASP1* Gene Expression in Vaginal Samples

The cellular fractions of vaginal samples were lysed using TRIzol (Life Technologies, Monza, Italy). Total RNA was extracted and immediately retro-transcribed by using the Moloney murine leukemia virus reverse transcriptase reaction (M-MLV RT), as described in the manufacturer’s instructions. cDNA concentration was determined using a spectrophotometer. Human *GADPH*, *NLRP3*, *CASPASE1* (*CASP1*) and *C. albicans ACT1*, *SAP2*, *SAP5*, *SAP6*, *KEX2*, *ECE1*, and *HWP1* gene expression were detected by using primers reported in Table 1 and showing similar capacity and efficiency in detecting expression of the above genes. Primers for detected *KEX2*, *ECE1*, and *HWP1* genes were designed using SnapGene software, and the other primers were reported elsewhere (Naglik et al., 2008; Li et al., 2016; Awad et al., 2017). Before use, all primers were checked and analyzed with Oligo Analyzer 3.1 IDT and BLAST against all available *C. albicans* genome sequences to exclude they fall within hypervariable gene regions.

**Table 1:** Primer sequences used for detection of *C. albicans ACT1*, *SAP2*, *SAP5*, *SAP6*, *KEX2*, *ECE1*, and *HWP1* genes and human *GADPH*, *NLRP3*, and *CASP1* genes.

Genes name	Sequences of forward primers (5′-3′)	Sequences of reverse primers (5′-3′)
*ACT1*	GACAATTTCTCTTTCAGCACTAGTAGTGA	GCTGGTAGAGACTTGACCAACCA
*SAP2*	TCCTGATGTTAATGTTGATTGTCAAG	TGGATCATATGTCCCCTTTTGTT
*SAP5*	CATTGTGCAAAGTAACTGCAACAG	CAGAATTTCCCGTCGATGAGA
*SAP6*	CCTTTATGAGCACTAGTAGACCAAACG	TTACGCAAAAGGTAACTTGTATCAAGA
*KEX2*	AGGAAGTGGTCGTCGTCGTA	ACGCCTGGTGTCGGAATCAT
*ECE1*	CAGAGCTGTTGACACAGCC	CACCAGTGGCAACACGAGC
*HWP1*	CCACAGGTAGACGGTCAAGG	CTGTTGTGGATAGTCACATGGC
*GADPH*	CGGATTTGGTCGTATTGGG	CTCGCTCCTGGAAGATGG
*NLRP3*	GGAGAGACCTTTATGAGAAAGCAA	GCTGTCTTCCTGGCATATCACA
*CASP1*	CCAGGACATTAAAATAAGGAAACTGT	CCAAAAACCTTTACAGAAGAATCTC

Real-time PCR (quantitative PCR) was performed in 96-well PCR plates (Thermo Scientific, Waltham, MA USA) using SYBR green (BioRad, Milan, Italy). For real-time PCR reaction, 200 ng of cDNA was used. All samples were measured in triplicates. The expression levels of *SAP2*, *SAP5*, *SAP6*, *KEX2*, *ECE1*, *HWP1*, *NLRP3*, and *CASP1* were calculated by comparative Ct method (2^−∆Ct^ formula) after normalization with *ACT1* for *C. albicans* genes and *GADPH* for human genes (Ginzinger, 2002; Bostanci et al., 2011; Dang et al., 2015; Roselletti et al., 2017). Amplification conditions were the same for all genes examined: genes: 3 min at 95°C, 40 cycles of 10 s at 95°C, and 30 s at primer specific temperature. The experiments were performed using Applied Biosystems 7300 (Thermo Scientific). Over expression of relevant genes was defined as >2 times the value of housekeeping gene expression for vaginal sample.

### Strains, Media, and Culture Conditions for Mouse Infection

Two *C. albicans* strains (hereafter designated as CA-105 and CA-67), isolated from two VVC patients enrolled in this study were used. The cultures were maintained by serial passages on YPD agar. The yeast cells were harvested by suspending a single colony in saline, washed twice, counted in a hemocytometer, and adjusted to the desired concentration for each experiment.

### Mice

Female CD1 mice obtained from Charles River (Calco, Italy) were used at 4–6 weeks of age. Mice were allowed to rest for 1 week before starting the experiment; by that time, the animals were roughly 5–7 weeks old. Animals were used under specific-pathogen free conditions that included testing sentinels for unwanted infections that were not detected, according to the Federation of European Laboratory Animal Science Association standards.

### Experimental Infection Model

Mice were vaginally infected as previously described (Enjalbert et al., 2009; Pericolini et al., 2015, 2017). Mice were maintained under pseudoestrus condition by subcutaneous injection of 0.2 mg of estradiol valerate in 100 μl of sesame oil (Sigma-Aldrich) 2 days prior to infection and 1 day after infection. Mice anesthetized with 2.5–3.5 (v/v) isoflurane gas were infected with 10 μl of 2 × 10^9^ cell/ml of CA-105 or CA-67 strains. Cell suspensions were administered with a mechanical pipette inserted into the vaginal lumen, close to the cervix. To favor vaginal contact and adsorption of fungal cells, mice were held head down for 1 min following inoculation. Mice were then allowed to recover for 24 h, during which *Candida* infection was established. The vaginal pH was measured with vaginal swab by pH-Fix strips (Macherey-Nagel GmbH & Co. KG, Germany) in each mouse before (day −2) and after (day 0) subcutaneous injection of estradiol valerate and at the end of experiments (day +3).

### Colony-Forming Units Assay

At day +3 post-infection, mice were sacrificed and vaginal swab was performed under aseptic conditions. The swab was soaked in 220 μl of saline, vortexed vigorously for at least 1 min and removed. Fifty microliter of vaginal fluid or 100 μl of 10-fold serial dilutions were plated in Sabouraud glucose agar with chloramphenicol (SAB + C) plate and incubated at 37°C for 48 h. The fungal load was expressed as the number of colony-forming units (CFU) in 200 μl of saline. Twenty microliter of each vaginal swab were also stained with Haemacolor and examined by light microscopy (Olympus) for the presence of neutrophils and *C. albicans* morphology. Then, the remainder of vaginal swab (100 μl) was centrifuged at 10,000 rpm for 10 min, and the supernatant used for cytokine production while cellular fraction was tested for gene expression.

### IL-1β Production

The supernatants (see above) were collected and tested for IL-1β production by specific ELISA assays (eBioscence, San Diego, CA). Cytokine titers were calculated relative to standard curves.

### Quantitative Analysis of *SAP2* and *ECE1* Expression in Mouse Model

Cellular fraction of vaginal swab was lysed, total RNA extracted and retro-transcribed. cDNA concentration was determined and *C. albicans*, *ACT1*, *SAP2*, and *ECE1* were detected as described above. All samples were measured in triplicates, and cDNA quantities reported as 2^−ΔΔCt^ relative to the *Candida* at day 0 (inoculum) or as 2^−ΔCt^ relative expression.

### Statistical Analysis

GraphPad Prism 7.0 software was used to test the normal distribution and for all presented statistical analysis. Because the distribution of gene expression data in VVC was very wide and not normal, we initially used the non-parametric Spearman test to calculate the correlation coefficient *r*. However, when the data were subjected to log transformation, the curves took a normal distribution (except in the case of *NLRP3*/caspase-1 correlation), thus the log transform was also used to calculate a Pearson coefficient as second test confirmation as well as to report the widely distributed data in easily readable graphic format. The Pearson (log transform) and Spearman (original data) gave similar results in terms of *r* values and statistical significance, set at *p* < 0.05.

For CFU assay and IL-1β production in mice challenged with VVC isolates, the quantitative variables were compared by ANOVA and *post-hoc* comparisons were done with Bonferroni’s test. For gene expression, the quantitative variables were compared by means of Student’s two-tailed *t* test. Values of *p* < 0.05 were considered significant.

### Ethics Statement

The procedures involving the animals and their care were conducted in conformity with the national and international laws and policies. All animal experiments were performed in agreement with the EU Directive 2010/63, the European Convention for the Protection of Vertebrate Animals used for Experimental and other Scientific Purposes, and the National Law 116/92. The protocol was approved by Perugia University Ethics Committee for animal care and use (Comitato Universitario di Bioetica, permit number 795/2016-PR). All the animals were housed in the animal facility of the University of Perugia (Authorization number 34/2003A). Mice were acclimatized for a week before starting the experiments. Three mice were housed in each cage and were provided with food and water ad libitum. All efforts were made to minimize suffering during experiments.

## Results

### Transcription of *NLRP3* Inducers of *Candida albicans* in Vulvovaginal Candidiasis

We previously reported on a common, high expression of genes coding for virulence and immuno-inflammatory factors of *C. albicans*, the main causative agent of VVC (Roselletti et al., 2017). In this paper, we have hypothesized the above factors could be somewhat associated in their expression. For this, we preliminarily verified the existence of a biologically plausible association such as the one between *NLRP3* and *CASP1* expression in VEC of all 40 VVC patients so as to have a sort of positive control of our approach to statistical assessment. There was indeed a highly significant (*p* < 0.0001) positive correlation in the expression of the two genes (*r*: 0.60, Spearman test). Then, we asked whether *SAP2/SAP5/SAP6 and ECE1* expression correlated with *NLRP3* and/or with *CASP1* expression. The data showed that *SAP2* and *ECE1* expression significantly correlated, though with a moderate strength, to the *NLRP3* inflammasome expression, while *SAP6* had a borderline significance and *SAP5* no statistical significant correlation at all with *NLRP3*. Interestingly, all the above genes significantly correlated with Caspase-1 expression (data not shown and Supplementary Figure S1).

**Figure 1:**
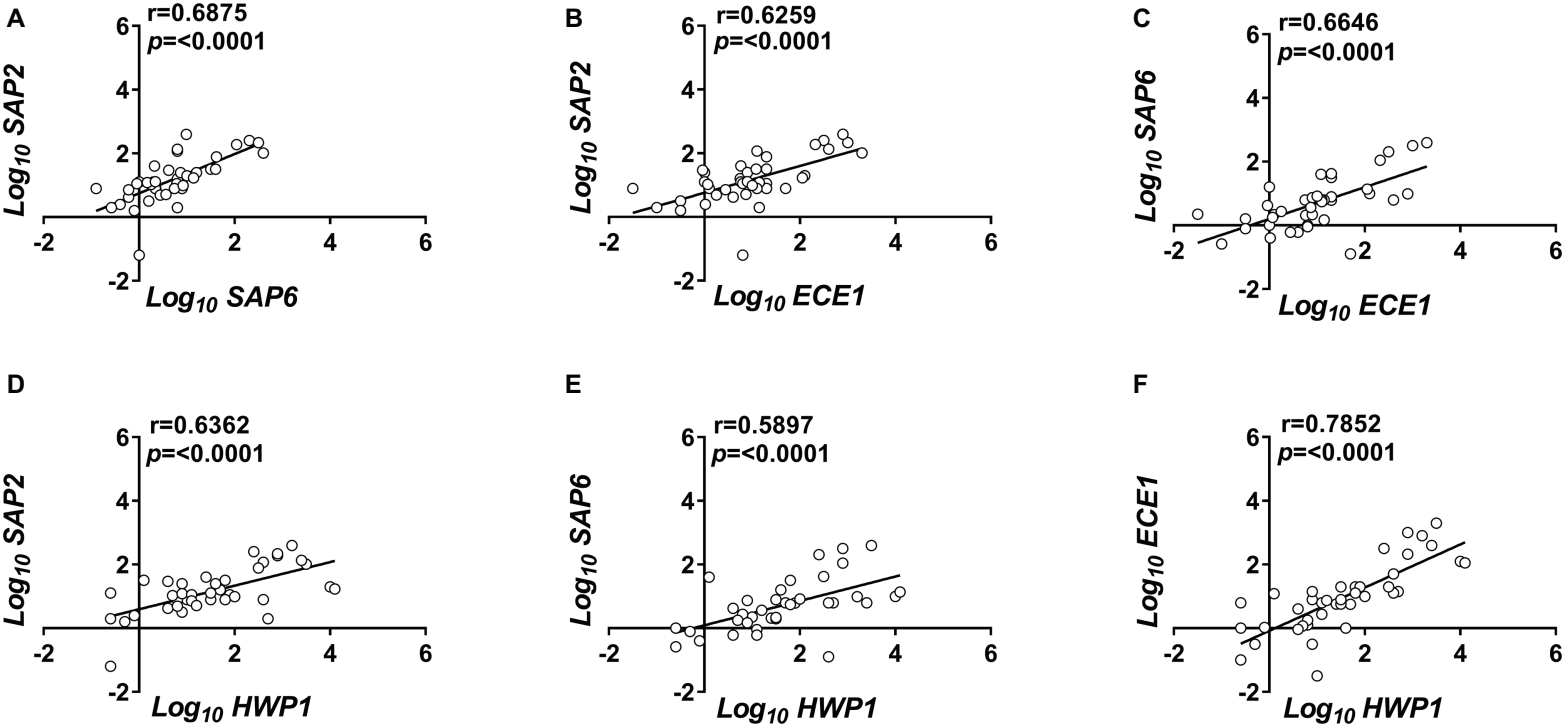
Correlated expression of *C. albicans* genes. Vaginal samples of symptomatic women were centrifuged at 3,000 rpm for 10 min, then cellular fractions were lysed, and total RNA was extracted and retro-transcribed to cDNA. **(A–F)** The expression levels of *SAP2*, *SAP6*, *ECE1*, and *HWP1* genes were calculated by comparative Ct method (2^−∆Ct^ formula) after normalization with *ACT1* gene. The results reported are from triplicates samples from 40 VVC subjects. Linear regression lines are shown. Pearson correlation (*r*) and statistical significance are indicated in each panel.

We then asked how *SAP2*, which codes for a typical yeast form-associated protein, was expressed in relation to *SAP6* and *ECE1*, both of which code for typical hyphae-associated proteins (Naglik et al., 2003; Peters et al., 2014). To strengthen the above verification, we included *HWP1* which codes for a hyphae-associated adhesin, the *HWP1* (Tsuchimori et al., 2000). The data are shown in Figure 1. *SAP2* and *SAP6* transcription proved to be significantly correlated and both appear to be significantly associated with *ECE1* and *HWP1* expression (Figures 1A–E). Particularly strong was the correlation between *ECE1* and *HWP1* (*r* = 0.79; Figure 1F). For the whole VVC population under study, the ratio *SAP2/ECE1* was slightly above the unit (mean ± SEM: 1.07 ± 0.135).

Since *SAP*, *HWP1*, and *ECE1* proteins are all downstream dependent of the activity of a protease encoded by *KEX2* (Tsuchimori et al., 2000; Newport et al., 2003), the afore-mentioned correlations could be only indirect (spurious) and actually due to a correlation with *KEX2* expression. Therefore, we tested whether *SAP2*, *ECE1*, and *HWP1* transcription was correlated to the expression of *KEX2* gene. All three genes were indeed significantly associated to *KEX2* expression; however, the correlation was generally moderate to weak (*r* < 0.6), particularly with *SAP2* (*r* = 0.4009), suggesting that the correlated expression of the virulence factors is unlikely to be entirely attributable to their correlation with *KEX2* expression (Supplementary Figure S2).

### Candida Cells Morphology in Acute Vulvovaginal Candidiasis

**Figure 2:**
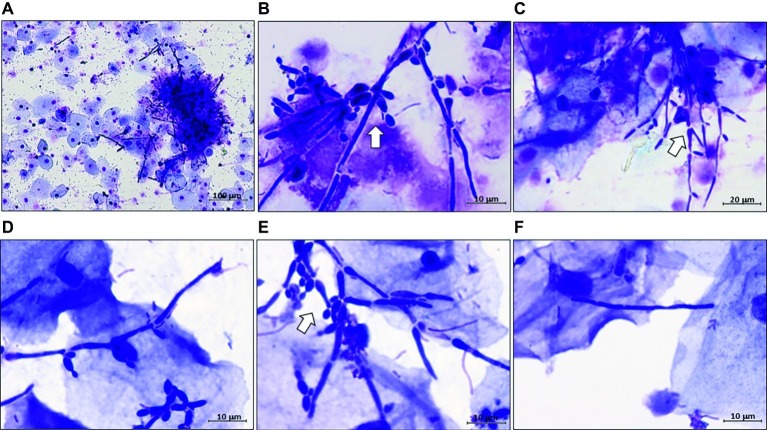
Representative images of *C. albicans* polymorphism in vaginal samples of VVC patients. All VVC samples were microscopically examined after Haemacolor staining for the presence of yeast, hyphal, and pseudo-hyphal forms of *C. albicans*. Representative images are from five different women, randomly selected. Original magnifications: **(A)** 100×, **(B)** 1,000×, **(C)** 400×, **(D–F)** 1,000×. **(A,B)** are two images, at different magnifications, from the same VVC patient. Note in panel **(A)**, a complex cluster of hardly distinguishable forms, and in panel **(F)**, a single filament that could be annotated as hyphal cell but remains of doubtful assignment. Arrows point to complex pseudohyphal forms.

The correlation data regarding *SAP2* with *ECE1*, *HWP1*, and *SAP6* were contrary to expectations. Yeast and filamentous forms of growth are generally both present in the vaginal samples of our VVC patients (Roselletti et al., 2017). However, their proportions vary extensively from patient to patient, that would contrast the positive correlation in the expression of these genes in the whole VVC population if the popular associations *SAP2*-yeast, *ECE1*, SAP6, and *HWP1*-hyphae were valid. To obtain further insight into this aspect, we re-assessed quantitatively the different forms of growth present in the vaginal samples of our patients. All samples were examined and seen to contain yeast and the expected filaments, sometimes in clusters of poorly distinguishable growth forms. This despite, more than 90% of the filamentous forms, whichever their elongation and clustering, had constricted septa and lateral buds, being thus ascribable to pseudohyphae (pseudomycelia) rather than true hyphae (Sudbery et al., 2004) (see Figure 2 and Supplementary Figure S3).

### *Candida albicans* Morphology and *SAP2/ECE1* Expression in Mouse Vaginal Infection With Vulvovaginal Candidiasis Isolates

**Figure 3:**
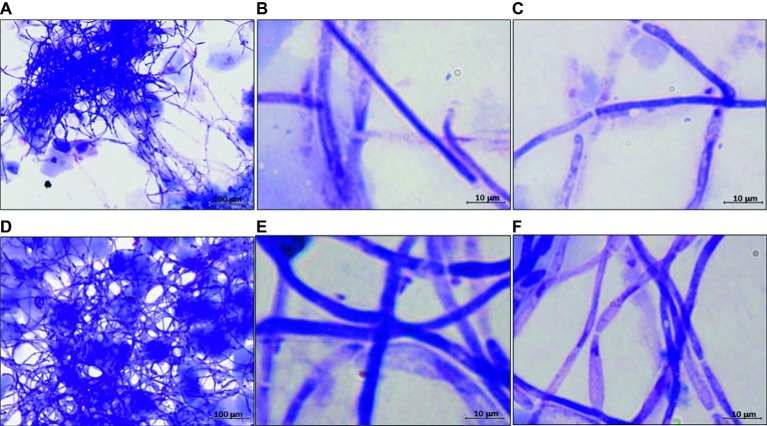
Representative images of hyphal development of *C. albicans* in MVC. Panels **(A–C)** are from mice challenged with CA-105 VVC strain. Panels **(D–F)** are from mice challenged with CA-67 VVC strain. Panels **(A,D)**: 100×; panels **(B–F)**: 1,000×.

**Figure 4:**
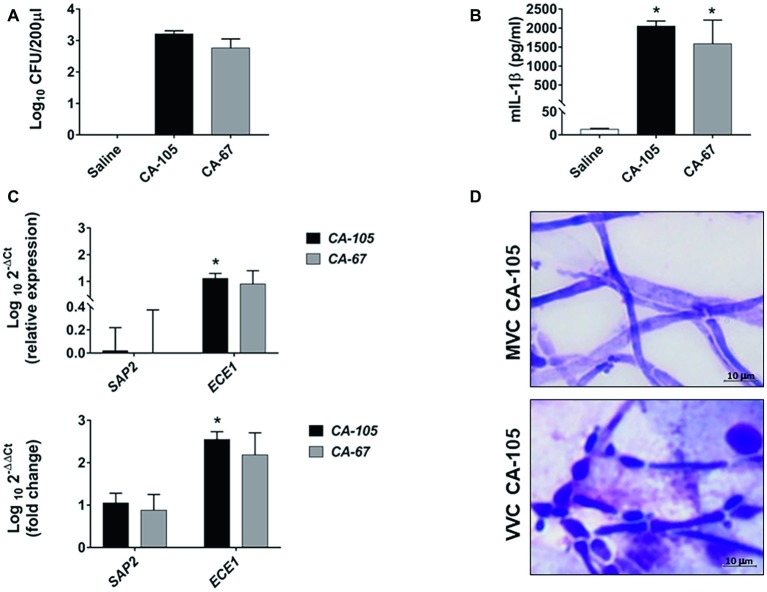
Fungal burden, cytokine production, *SAP2* and *ECE1* gene expression, and *C. albicans* morphology in CD1 mice. Panels **(A,B)** CD1 female mice were infected with two clinical isolates of *C. albicans* (CA-105 and CA-67). Vaginal swab was taken from each mouse 3 days post-infection. Fungal burden was evaluated by CFU counts **(A)**, while IL-1β production was quantified by ELISA **(B)**. Data are from one experiment with *n* = 3 mice/group. The graphs show the mean ± SEM and statistical significance was evaluated with ANOVA test + Bonferroni. ^*^p < 0.05 infected mice vs. uninfected mice. Panel **(C)** The cellular fractions from the vaginal swab were lysed, and total RNA was extracted and immediately retro-transcribed into cDNA. *SAP2* and *ECE1* genes were detected as described in “Materials and Methods” section. All samples were measured in triplicates and amplified cDNA quantities reported as 2^−ΔΔCt^ relative to the *Candida* at day 0 (inoculum) (graph below) or as 2^−ΔCt^ fold expression (graph above). Data for *SAP2* and *ECE1* genes are the mean ± SEM from one experiment with *n* = 3 mice/group, and statistical significance was evaluated with Student *t* test. ^*^p < 0.05 *SAP2* vs. *ECE1* expression. Panel **(D)** Comparative images of CA-105 morphology in MVC (above) and in VVC (below) are shown (original magnification 1,000×, scale bar = 10 μm).

The data of the previous section led us to assess *C. albicans* growth forms and gene expression in a model of mouse vaginal candidiasis (MVC) after challenge with two fungal isolates from VVC subjects (CA-105 and CA-67). In this experiment, the vaginal pH was measured before and after pseudoestrus induction, as well as on day 3 post-challenge, that is known to be the day of optimal fungus colonization. We focused upon expression of *SAP2* and *ECE1* that previous studies demonstrated to play an important, though likely distinct role in the above model (Roselletti et al., 2017; Richardson et al., 2018a,b). The results are reported in Figures 3, 4. As shown in Figure 3 (growth form) and panel A (CFU) of Figure 4, both isolates were able to colonize mouse vagina as pure hyphal forms, represented by long, parallel-sided filaments with total absence of yeast cells. On the other hand, as shown in panel B of Figure 4, both isolates were able to cause vaginal inflammation as inferred by the high production of *NLRP3*-dependent cytokine IL-1β. As also expected from previous studies (Bruno et al., 2015; Pericolini et al., 2015), there was a high level of *ECE1* and a relatively low one of *SAP2* expression on day 3 post-infection (panel C). Panel D of Figure 4 shows a direct comparison of pseudohyphal (VVC) and hyphal (MVC) cells of the same vaginal isolate (CA-105) of *C. albicans*. The mouse vaginal pH was always close to neutrality (6.8–7.2) at each time examined.

Table 2 recapitulates the differences between VVC and MVC as assessed in this study.

**Table 2:** Some differences between VVC caused by *C. albicans* and experimental vaginal infection by this fungus in mice (MVC).

Disease	Vaginal pH	Dominant form of growth	Gene expression (log)^a^	
			*SAP2*	*ECE1*
VVC	4.5–5.0	Pseudohyphae^b^	1.17 ± 0.11	0.97 ± 0.16
MVC	6.8–7.2	True hyphae^c^	0.96 ± 0.22	2.36 ± 0.29^d^

a *Mean ± SEM (log relative expression) of 40 VVC cases and mean ± SEM log fold expression of 6 mice (three determinations each) challenged with CA-105 (3 mice) and CA-67 (3 mice)*;

b *Yeast cells are present*;

c *Yeast cells are absent*;

d *p < 0.05, Student’s *t* test, two tailed, comparing *SAP2* with *ECE1* in MVC*.

## Discussion

Recent findings from experimental mouse models of vaginal infection by *C. albicans* (MVC) provide direct evidence that both secretory aspartyl proteinases (Sap, in particular *SAP2* and *SAP6*) and *ECE1*-derived toxin, candidalysin, can trigger the signaling cascade causing *NLRP3* inflammasome activation in VEC (Bruno et al., 2015; Pericolini et al., 2015; Richardson et al., 2018b; Rogiers et al., 2019). However, *SAP2*/*SAP6* and candidalysin have been reported to act optimally at different times of infection (1 and 3 days post-fungal challenge, respectively) (Bruno et al., 2015; Pericolini et al., 2015). In addition, *SAP2* is typically associated with the yeast form of *C. albicans*, whereas candidalysin production is strictly dependent on hyphal formation (Richardson et al., 2018b) and does not require Sap activity (Richardson et al., 2018a). This suggests that in MVC, *SAP2*, and *ECE1* are expressed independently of each other and their products do not interact in their pro-inflammatory conducive pathways.

We have previously shown that *NLRP3* inflammasome activation is also a hallmark of vaginal inflammatory disease in VVC patients. In contrast to *C. albicans* carriers, asymptomatic subjects, VVC patients are characterized by the concurrent, high expression level of fungal genes coding for inflammasome inducers and virulence genes such as *SAP2/SAP6*, *ECE1*, and *HWP1* (Pericolini et al., 2015; Roselletti et al., 2017). We show here that the expression of these genes is positively correlated, with a highly significant statistical confidence, though with a generally moderate strength. Whether the correlation is direct or indirect (referring to a third, unknown factor) is unclear, although a weak correlation between *SAP2*, *HWP1*, and *ECE1* with *KEX2* expression has been found. In addition, *SAPs*, *ECE1*, and *HWP1* expression appears to be substantially correlated with Caspase-1, a critical component of VEC inflammation machinery.

Our cytological observations show that the presence of filamentous forms resembling true hyphae is very limited, if any, in the vaginal exudate of VVC patients, where the dominant filaments appear to be the pseudohyphal ones, in a patient-dependent variable proportion with yeast cells. Thus, the whole fungal morphological scenario and the correlations in gene expression in VVC appears to be typical of human infection, and incompatible with the popular assumption that *SAP2* is a yeast, and *SAP6*, *ECE1*, and *HWP1* are hyphae-associated in their expression. Although usually confused with true hyphae and invariably defined as filamentous forms in many VVC reports, pseudohyphae and hyphae have remarkable differences in morphology, structure, mechanism of cell division, and metabolism (Sudbery et al., 2004; Ahmed et al., 2018). Unfortunately, pseudohyphae remain poorly investigated.

Among the biological and physico-chemical differences between human and mouse vagina, a critical factor to explain our data on fungal morphology and gene-expression could be the vaginal pH that exerts indeed strong regulation of gene expression and morphogenesis of *C. albicans* (Buffo et al., 1984; Saporito-Irwin et al., 1995; De Bernardis et al., 1998; Davis et al., 2000).

VVC is characterized by an acidic pH (range 4.5–5), as confirmed here and in a previous study (Roselletti et al., 2019). In *in vitro* cultures of *C. albicans*, these acidic pH values are usually permissive of yeast, not hyphal growth. At the relatively high CO_2_ concentration and metabolite-rich environment (as in the vagina), the fungus can also grow under filamentous forms but these are dominantly pseudo-hyphae (Jakab et al., 2016). Acidic pH causes cell wall remodeling with un-masking of beta-glucan (Sherrington et al., 2017), a fact that has been previously demonstrated in VVC patients (Pericolini et al., 2018; Roselletti et al., 2019). Beta-glucan is a strong trainer of innate immunity and *NLRP3* inflammasome activation (Camilli et al., 2018) that is considered of mechanistic relevance in vaginal candidiasis (Cassone, 2018). In contrast, and despite some different data (Ganesan and Kadalmani, 2016), the pH of mouse vagina has been reported to be around neutrality (Yano and Fidel, 2011). We have here measured the vaginal pH of mice experimentally infected by two VVC isolates and confirm it is indeed close to neutrality, both before and after pseudoestrus induction and infectious challenge, as well. The dominant, if not exclusive form of growth of these isolates in the mouse vagina was the expected true hyphal one, and it was particularly vigorous in our experimental setting. Of potential mechanistic importance, the *ECE1/SAP2* expression ratio was in infected mice largely in favor of *ECE1*, whereas it was slightly above 1 in our VVC population, suggesting for a potentially different impact of *SAP2* and candidalysin in human vs. mouse infection.

This study has several limitations. First of all, correlation does not mean causation. Second, gene expression cannot exactly predict amount and activity of the related protein products that ultimately trigger inflammasome activation. Third, the number of VVC cases is relatively small. Fourth, the examined growth forms of the fungus are those typically released in the vaginal exudate and might not be entirely representative of those invading the epithelial tissue. It should be considered that the association of gene expression in VVC with yeast/pseudohyphal cells, though plausible with the present clinical and experimental evidence, remains indirect. Despite the above limitations, our data clearly show that pseudohyphal cells are the dominant filamentous growth form of *C. albicans* in VVC exudate, whereas true hyphal cells dominate in the vaginal exudate in MVC. The use of VVC isolates for mouse infection rules out that the different growth forms in VVC and MVC could be due to *C. albicans* strain difference. Altogether, our data suggest that pseudohyphal cells may exert a specific role in VVC causation. In this line, worthy of mechanistic consideration is the remarkable difference between VVC and MVC in the expression of *SAP2* and *ECE1*, two recognized *NLRP3* inflammasome activators. It appears that under yeast/pseudohyphal form, probably fostered by the acidic pH of human vagina, the fungus is well equipped to-express, in a correlated fashion, several genes coding for *NLRP3* inducers and fungus virulence suggesting that multiple fungal components could directly cooperate to determine VVC.

Overall, the differences between VVC and MVC, some of which are summarized in Table 2, would argue against the mouse model being a real proxy of human disease. In particular, we invite careful consideration of the differential properties of *C. albicans* growth forms, when translating from MVC to VVC data dealing with expression of *C. albicans* virulence factors, inducers of *NLRP3* inflammasome activation and vaginal inflammation.

## Data Availability Statement

All data supporting the conclusions of this manuscript are fully available upon reasonable request.

## Ethics Statement

The studies involving human participants were reviewed and approved by Local Ethical Committee CEAS (Comitato Etico delle Aziende Sanitarie, Umbria, Italy) approval was received for the whole study (VAG1 n. 2,652/15). Written informed consent for participation was not required for this study in accordance with the national legislation and the institutional requirements. The animal study was reviewed and approved by Perugia University Ethics Committee for animal care and use (Comitato Universitario di Bioetica, permit number 795/2016-PR).

## Author Contributions

AC and AV conceived and designed the experiments. ER, SP, SS, and CM performed the experiments and analyzed the data. AC wrote the paper. AV, JS, and AC revised the paper. All authors read and approved the final manuscript.

### Conflict of Interest

The authors declare that the research was conducted in the absence of any commercial or financial relationships that could be construed as a potential conflict of interest.

## Supplementary Material

SUPPLEMENTARY FIGURE S1 Correlation between *C. albicans* genes and human EC inflammasome genes. Vaginal samples of symptomatic women were centrifuged at 3000 rpm for 10 min, then cellular fractions were lysed, and total RNA was extracted and retro-transcribed in cDNA. The expression levels of *SAP2*, *SAP5*, *SAP6*, *ECE1*, and *CASP1* genes were calculated by comparative Ct method (2^−∆Ct^ formula) after normalization with *ACT1* for *C. albicans* genes or *GADPH* for human genes. The results reported are from triplicates samples for 40 symptomatic women. Linear regression lines are shown. Pearson correlation (*r*) or Spearman correlation (*r*) and statistical significance are indicated in each panel.

SUPPLEMENTARY FIGURE S2 Correlation between *KEX2* and *SAP2*, *ECE1*, and *HWP1* gene expression. Vaginal samples of symptomatic women were centrifuged at 3000 rpm for 10 min, then cellular fractions were lysed, and total RNA was extracted and retro-transcribed in cDNA. The expression levels of KEX2, *SAP2*, *ECE1*, and *HWP1* genes were calculated by comparative Ct method (2^−∆Ct^ formula) after normalization with *ACT1* gene. The results reported are from triplicates samples for 40 symptomatic women. Linear regression lines are shown. Pearson correlation (*r*) and statistical significance are indicated in each panel.

SUPPLEMENTARY FIGURE S3 Images of VVC samples from different subjects illustrating the polymorphism of *C. albicans* cells with dominance of pseudohyphal elements in different aggregates. Magnification is indicated in each image.
