# Supplementary figures and images for: A Role for Yeast/Pseudohyphal Cells of Candida albicans in the Correlated Expression of NLRP3 Inflammasome Inducers in Women With Acute Vulvovaginal Candidiasis
Source: Front Microbiol. 2019 Nov 15;10:2669. doi: 10.3389/fmicb.2019.02669 (PMC6873873; doi:10.3389/fmicb.2019.02669)

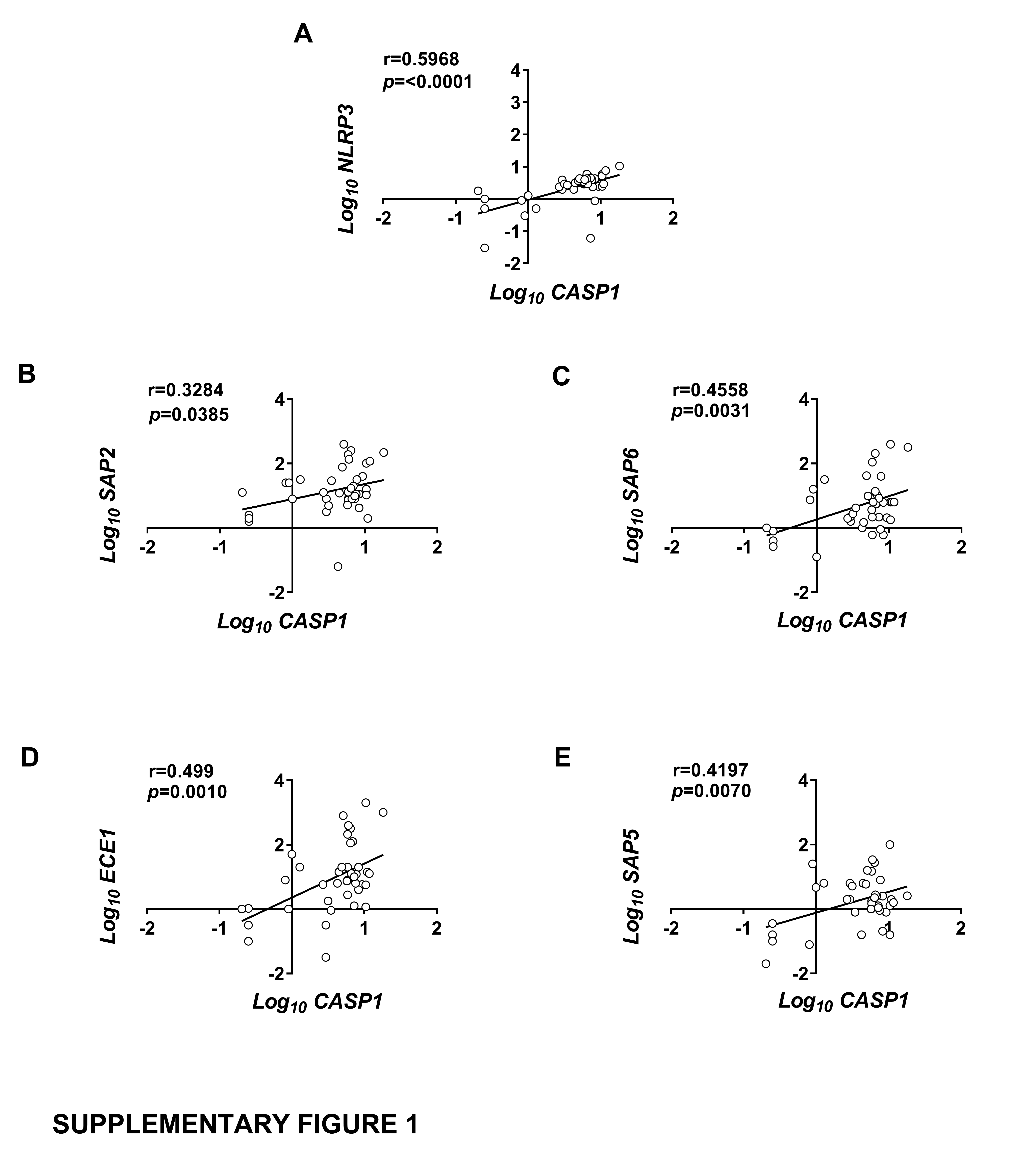

Supplement: SUPPLEMENTARY FIGURE S1 — Correlation between C. albicans genes and human EC inflammasome genes. Vaginal samples of symptomatic women were centrifuged at 3000 rpm for 10 min, then cellular fractions were lysed, and total RNA was extracted and retro-transcribed in cDNA. The expression levels of SAP2, SAP5, SAP6, ECE1, and CASP1 genes were calculated by comparative Ct method (2−∆Ct formula) after normalization with ACT1 for C. albicans genes or GADPH for human genes. The results reported are from triplicates samples for 40 symptomatic women. Linear regression lines are shown. Pearson correlation (r) or Spearman correlation (r) and statistical significance are indicated in each panel. [file Image_1.TIF]

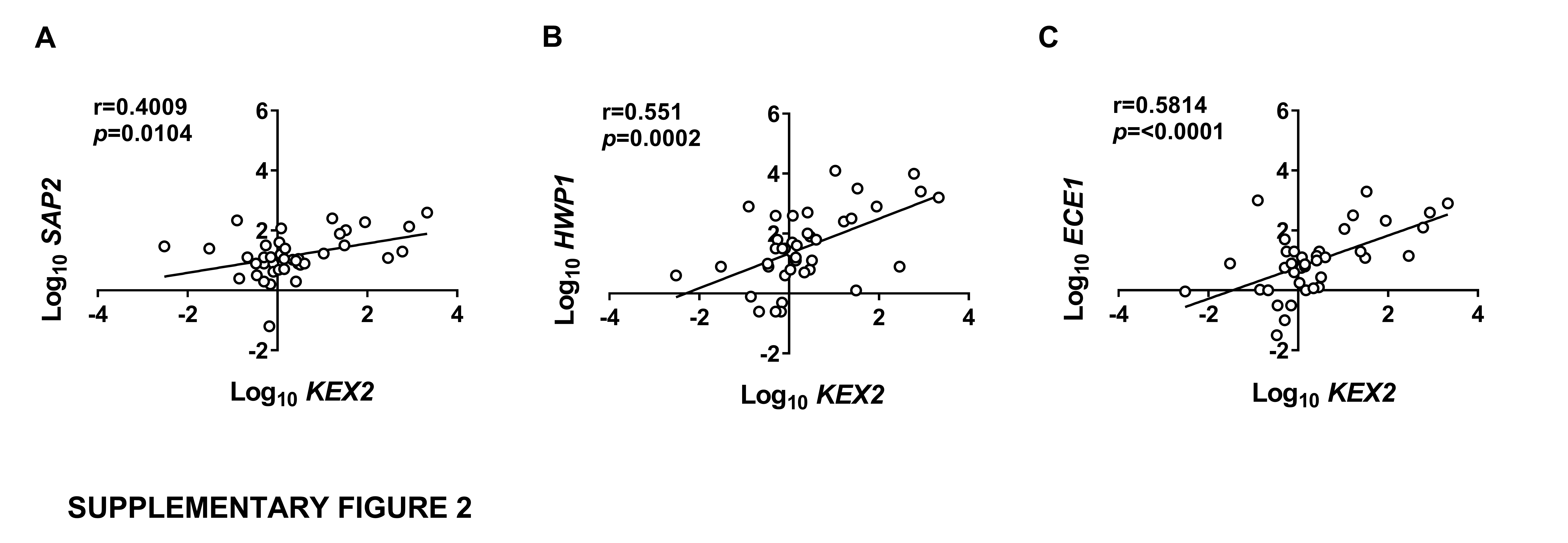

Supplement: SUPPLEMENTARY FIGURE S2 — Correlation between KEX2 and SAP2, ECE1, and HWP1 gene expression. Vaginal samples of symptomatic women were centrifuged at 3000 rpm for 10 min, then cellular fractions were lysed, and total RNA was extracted and retro-transcribed in cDNA. The expression levels of KEX2, SAP2, ECE1, and HWP1 genes were calculated by comparative Ct method (2−∆Ct formula) after normalization with ACT1 gene. The results reported are from triplicates samples for 40 symptomatic women. Linear regression lines are shown. Pearson correlation (r) and statistical significance are indicated in each panel. [file Image_2.TIF]

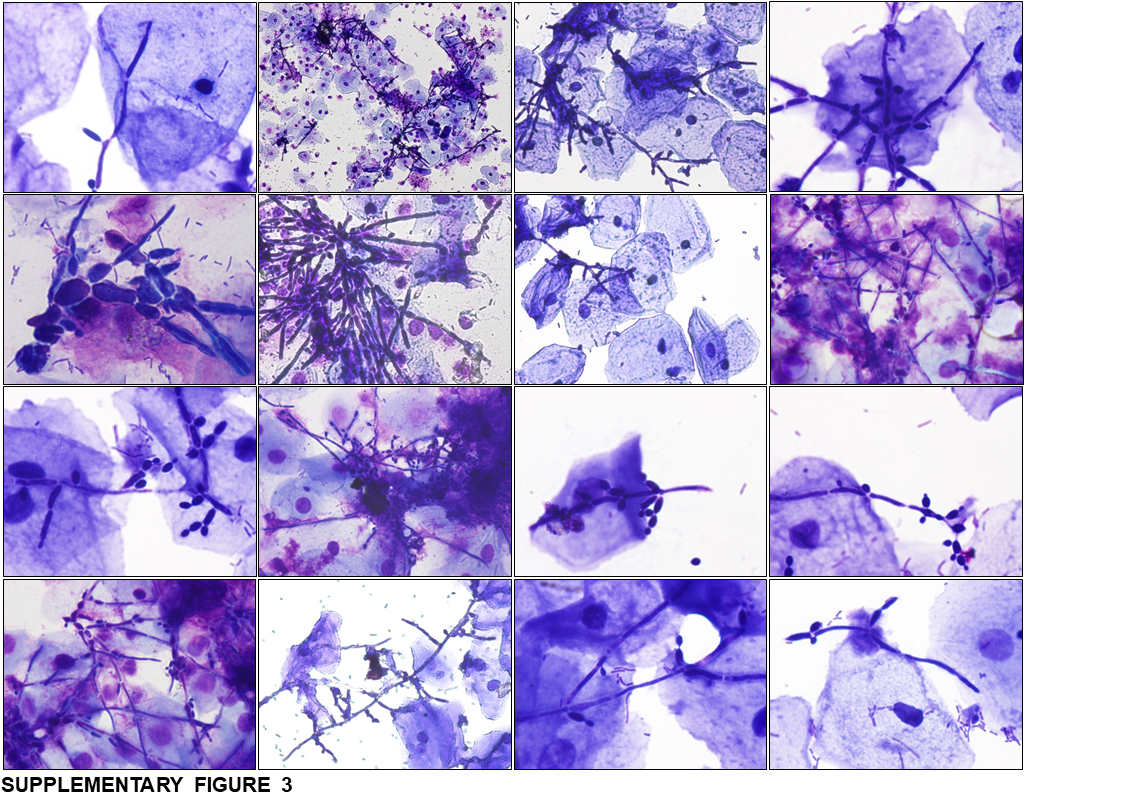

Supplement: SUPPLEMENTARY FIGURE S3 — Images of VVC samples from different subjects illustrating the polymorphism of C. albicans cells with dominance of pseudohyphal elements in different aggregates. Magnification is indicated in each image. [file Image_3.TIF]
